# VAST: Vertebral Artery Stenting Trial. Protocol for a randomised safety and feasibility trial

**DOI:** 10.1186/1745-6215-9-65

**Published:** 2008-11-24

**Authors:** A Compter, HB van der Worp, WJ Schonewille, JA Vos, A Algra, TH Lo, WPThM Mali, FL Moll, LJ Kappelle

**Affiliations:** 1 Department of Neurology, Rudolf Magnus Institute for Neuroscience, University Medical Centre Utrecht, HP G 03.228, PO Box 85500, 3508 GA Utrecht, The Netherlands; 2 Department of Neurology, St. Antonius Hospital, PO Box 2500, 3430 EM Nieuwegein, The Netherlands; 3 Department of Radiology, St. Antonius Hospital, PO Box 2500, 3430 EM Nieuwegein, The Netherlands; 4 Julius Center for health Sciences and Patient Care, University Medical Centre Utrecht, HP STR 6.131, PO Box 85500, 3508 GA Utrecht, The Netherlands; 5 Department of Radiology, University Medical Centre Utrecht, HP E01.132, PO Box 85500, 3508 GA Utrecht, The Netherlands; 6 Department of Surgery, University Medical Centre Utrecht, HP G04.130, PO Box 85500, 3508 GA Utrecht, The Netherlands

## Abstract

**Background:**

Twenty to 30 percent of all transient ischaemic attacks and ischaemic strokes involve tissue supplied by the vertebrobasilar circulation. Atherosclerotic stenosis ≥ 50% in the vertebral artery accounts for vertebrobasilar stroke in at least one third of the patients. The risk of recurrent vascular events in patients with vertebral stenosis is uncertain and revascularisation of vertebral stenosis is rarely performed. Observational studies have suggested that the risk of subsequent stroke or death in patients with vertebrobasilar ischaemic events is comparable with that in patients with carotid territory events. Treatment of vertebral stenosis by percutaneous transluminal angioplasty has been introduced as an attractive treatment option. The safety and benefit of stenting of symptomatic vertebral stenosis as compared with best medical therapy alone remains to be elucidated in a randomised clinical trial.

**Study objectives:**

The primary aim of the Vertebral Artery Stenting Trial (VAST) is to assess whether stenting for symptomatic vertebral artery stenosis ≥ 50% is feasible and safe. A secondary aim is to assess the rate of new vascular events in the territory of the vertebrobasilar arteries in patients with symptomatic vertebral stenosis ≥ 50% on best medical therapy with or without stenting.

**Design:**

This is a randomised, open clinical trial, comparing best medical treatment with or without vertebral artery stenting in patients with recently symptomatic vertebral artery stenosis ≥ 50%. The trial will include a total of 180 patients with transient ischaemic attack or non-disabling ischaemic stroke attributed to vertebral artery stenosis ≥ 50%. The primary outcome is any stroke, vascular death, or non-fatal myocardial infarction within 30 days after start of treatment. Secondary outcome measures include any stroke or vascular death during follow-up and the degree of (re)stenosis after one year.

**Discussion:**

Improvements both in imaging of the vertebral artery and in endovascular techniques have created new opportunities for the treatment of symptomatic vertebral artery stenosis. This trial will assess the feasibility and safety of stenting for symptomatic vertebral artery stenosis and will provide sufficient data to inform a conclusive randomised trial testing the benefit of this treatment strategy. The VAST is supported by the Netherlands Heart Foundation (2007B045; ISRCTN29597900).

## Background

Twenty to 30 percent of all transient ischaemic attacks (TIA's) and ischaemic strokes involve tissue supplied by the vertebrobasilar (VB) circulation [1,2]. The vertebral and basilar arteries feed the brain stem, cerebellum, and thalamus, and in most people the posterior temporal and occipital lobes as well. In a large American registry of posterior circulation stroke, 22% of the patients with VB TIA or stroke had a poor functional outcome at 30 days [3]. However, some forms of VB ischaemia have a more relentless course. Occlusion of the basilar artery, for example, is associated with death or dependence in almost 80% of the cases [4].

Common causes of VB ischaemia are embolism from the heart or large arteries, or small-vessel disease [2]. In the above-mentioned registry, 32% of the ischaemic events were presumed to be caused by haemodynamic mechanisms [3]. Atherosclerotic stenosis ≥ 50% at the origin of the vertebral artery or in its intracranial course were both found in about one third of the patients.

In contrast to carotid stenosis, there has been little systematic research into the prognosis and the prevention of recurrent vascular events in patients with vertebral artery stenosis. In the carotid surgery trials, patients with ≥ 50% symptomatic carotid stenosis randomised to medical treatment alone had a 5-year risk of ipsilateral carotid ischaemic stroke of 21.2% (95% CI, 18.8 – 23.6) [5]. Similar data are not available for symptomatic vertebral artery stenosis. The best available data come from a systematic review of all published studies of prognosis after VB TIA or minor stroke, in which the risk of subsequent stroke or death in patients with VB events was similar to the risk in patients with carotid territory events [1].

In patients with carotid stenosis of 50% or greater, carotid endarterectomy (CEA) leads to an absolute reduction in the 5-year cumulative risk of ipsilateral carotid ischaemic stroke and any stroke or death within 30 days after surgery of 8.5% (95% CI, 5.6 – 11.3) [5]. Two large randomised clinical trials are currently studying carotid stenting as an alternative to CEA. Based on large case series, it is hypothesized that the trials will demonstrate the equivalence of carotid stenting as compared with CEA. By contrast, the benefit of revascularisation of vertebral stenosis is fully uncertain. Surgery to this artery is difficult due to poor access and is not considered in most centres [6]. In the last decade, treatment of vertebral stenosis by percutaneous transluminal angioplasty, usually with stent placement, has been introduced as an attractive alternative to surgery.

The endovascular access to the vertebral artery is relatively easy and the procedure can be performed without general anaesthesia, enabling continuous neurological monitoring of the patient. However, the procedure also has disadvantages. The major risk of endovascular treatment is dislodgement and distal embolisation of plaque and thrombotic debris, which may lead to stroke. In a recent review of reports on 331 endovascular treatments of vertebrobasilar stenosis, the 30-day risk of TIA, stroke, or death was 6.4% [6]. In another review of stenting procedures for vertebral or basilar artery stenosis reported up to 2005, the periprocedural risk of stroke or death was 1.6% for proximal vertebral artery stenosis and 13.8% for distal VB stenosis [7]. After a mean follow-up of one year, restenosis had occurred in one quarter of the cases of proximal vertebral artery stenting. The difference in complication rates in the treatment of proximal versus distal vertebral artery stenosis is most likely the result of several factors. First, studies of distal VB artery stenosis included lesions of the basilar artery as well. In addition, the distal procedures were much more frequently performed in the acute phase of VB artery thrombosis, and antithrombotic treatment strategies were different. For this reason, there are no reliable data on stenting of the distal vertebral artery after the acute phase.

Case series are prone to publication bias and the percentages reported above may be different in daily clinical practice. The combined 30-day incidence of any stroke or death in randomised trials on carotid stenting versus CEA, was 8.2% (126/1492) after stenting [8]. Moreover, the interpretation of outcome results after vertebral stenting is complicated by the lack of data on the risk of recurrent stroke in patients with vertebral stenosis on best medical therapy alone. Most importantly, the only trial that has compared endovascular treatment with medical therapy in a randomised fashion, included just 16 patients [6,9].

In conclusion, stenting of vertebral artery stenosis appears a promising technique for the prevention of recurrent vascular events in the VB territory, but is still without a proven benefit. The procedure may be complicated by disabling stroke and early restenosis. Before widespread application, the procedure should be assessed in a large randomised trial. As a prelude to such a trial, the present study aims to determine the safety and feasibility of stenting for symptomatic vertebral artery stenosis.

## Study objectives

The present study aims to determine whether stenting for symptomatic vertebral artery stenosis ≥ 50% in patients with a recent transient ischaemic attack or minor disabling ischaemic stroke in the posterior circulation is feasible and safe. In addition, the rate of new vascular events in the territory of the vertebrobasilar arteries will be assessed in patients with symptomatic vertebral artery stenosis ≥ 50% who receive best medical treatment or best medical treatment combined with endovascular therapy. The results will serve to design a large and conclusive randomised clinical trial in which stenting plus best medical treatment will be compared to best medical treatment alone in patients with symptomatic vertebral artery stenosis.

## Study design

This is a randomised, open, multi-centre clinical trial with masked outcome assessment, comparing the combination of vertebral artery stenting and best medical treatment with best medical treatment alone in patients with a recently symptomatic stenosis of a vertebral artery of at least 50%. A total of 180 patients will be included. Follow-up will continue until one year after inclusion of the last patient.

Endovascular treatment will be performed by an experienced interventional radiologist with a track record of at least 50 interventions in the carotid or vertebral arteries in the last 5 years. The procedure will include percutaneous transluminal angioplasty followed by stenting. The type of stent and the use of a protection device will be left to the discretion of the interventionist. If stent placement is not feasible or deemed contra-indicated, angioplasty without stent placement will be performed. The procedure will be performed under local anaesthesia, with continuous monitoring of heart rhythm and blood pressure. All patients randomised to stenting will receive clopidogrel 75 mg daily starting at least five days before the procedure and continued for 30 days after the procedure. Patients not on clopidogrel the day before the procedure should be loaded with 300 mg clopidogrel at least six hours before stenting. Best medical treatment will be left to the discretion of the neurologist, but should include rigorous control of vascular risk factors, the use of antiplatelet agents, and the use of a statin.

### Enrolment criteria

Patients can be enrolled in the study if the following criteria have been met:

1. TIA or non-disabling ischaemic stroke of the posterior circulation;

2. symptoms occurred in the 180 days preceding randomisation;

3. possibility to perform stenting within two weeks after randomisation;

4. stenosis of the vertebral artery of 50% or greater, diagnosed by both duplex ultrasound and CT angiography (CTA), contrast-enhanced MR angiography (MRA), or conventional angiography, and presumed to be of atherosclerotic origin and accessible for endovascular treatment;

5. score on the modified Rankin scale ≤ 3 (independent in daily activities, although some help may be needed) [10];

6. age 40 years or older;

7. written informed consent.

Patients will be excluded from the study in case of

1. a potential cause of TIA or minor stroke other than stenosis in a vertebral artery (e.g. atrial fibrillation);

2. a vertebral artery stenosis caused by arterial dissection;

3. previous surgical or endovascular treatment of the stenosis;

4. a life expectancy shorter than three years;

5. another serious illness that may confound outcome assessment;

6. severe renal impairment, precluding contrast administration;

7. allergy to iodinated contrast agent;

8. pregnancy.

### Posterior cerebral artery TIA or infarct

In the majority of patients, blood supply via the posterior cerebral artery (PCA) to the occipital lobes is through the VB circulation. However, in a substantial portion of patients the PCA is fully supplied by the carotid artery instead of the VB arteries [11]. For this reason, we will assess the presence of an ipsilateral P1 segment of the PCA and of an ipsilateral posterior communicating artery on the initial MRA, CTA, or conventional angiography in all patients with (transient) ischaemia in the territory of the PCA. In case of uncertainty about the supply of the PCA, direction of flow in the posterior communicating artery will be assessed, for example by means of transcranial Doppler sonography. Of the patients with ischemia in the PCA territory only, those with an exclusive supply of the PCA via the carotid artery will be excluded.

### Randomisation

A total of 180 patients will be included in the study. Patients will be randomised to either the combination of vertebral artery stenting and best medical treatment or best medical treatment alone with use of a web-based randomisation system, which includes a minimisation algorithm [12]. Randomisation will be stratified by centre and location of the stenosis (origin vs. more distal parts of the vertebral artery).

### Outcome assessment

Clinical outcome assessment will be performed by three independent adjudicators on the basis of an anonymised written description of the outcome event and ancillary investigations. This description will be made by the research physician in such a way that the adjudicators will remain masked to the allocated treatment.

The primary outcome measure will be vascular death, non-fatal myocardial infarction, or non-fatal stroke (neurological deficit lasting longer than 24 hours for which no other cause than a stroke can be found) within 30 days after start of the treatment (see Appendix for definitions).

Secondary outcome measures will be vascular death, non-fatal myocardial infarction, or non-fatal stroke during follow-up. Other outcome measures include any stroke in the supply territory of the symptomatic vertebral artery during follow-up and the degree of stenosis of the symptomatic vertebral artery after one year, as assessed with both Duplex ultrasound and CT angiography.

### Data collection

At baseline, medical history (including clinical signs and symptoms, duration of the neurological deficit, number of attacks, previous cardiovascular events, previous cardiovascular treatments, vascular risk factors, and medication) will be assessed and clinical and neurological examination (including blood pressure) will be carried out. The baseline neurological and functional status will be assessed with the National Institutes of Health Stroke Scale (NIHSS) and with the modified Rankin Scale [10,13]. The vascular risk factors (hypertension, smoking, diabetes, lipid disorders) will be noted. Laboratory investigations will include complete blood count, erythrocyte sedimentation rate, C-reactive protein, and cholesterol.

The degree of stenosis in the vertebral artery will be assessed with duplex ultrasound and MRA, CTA, or conventional angiography. Except for duplex ultrasound, the degree of the stenosis will be calculated by dividing the residual lumen (N) by vessel diameter at a point distal to the stenosis where the normal vessel calibre has been restored (D), and applying the formula: (1 - N/D) × 100% = degree of stenosis [14]. To assess the location of stenosis, the vertebral artery is divided into four sections: V1-V3 form the extracranial vertebral artery and V4 forms the intracranial vertebral artery [15]. The type of plaque in the vertebral artery and the presence and degree of stenosis in other brain feeding arteries will be noted.

CT and MRI scans will be performed to investigate the presence and type of a cerebral infarct (supply territory of intracerebral artery, small vessel disease, large vessel disease)

### Follow-up

Follow-up visits will be performed at one day and at 1, 6, and 12 months after stenting (or randomisation in the conservative treatment group) and every year thereafter. The close-out visit of each patient will be scheduled one year after randomisation of the last patient.

Follow-up data to be obtained at one day and after 1, 6 and 12 months, every year thereafter, and at close out will include:

1. the occurrence and type of vascular event;

2. complications associated with the endovascular procedure;

3. medication;

4. blood pressure;

5. score on the modified Rankin Scale;

6. the degree of (re)stenosis assessed with duplex ultrasound and CTA will be assessed after one year and following an ischaemic stroke.

### Statistical considerations

With 90 patients in the endovascular intervention arm, a complication rate of 7.8% (7 events) would yield a 95% confidence interval from 3.2 to 15.4%. The estimate of 7.8% is comparable to that of 8.2% for carotid stenting [8]. As the number of patients receiving angioplasty without stent placement is expected to be extremely small, these patients will be analysed together with the patients who have received a stent.

Inclusion of a total of 90 patients in the medical arm during three years and one final year of follow up for these patients will provide 225 patient years of follow up. This number of patient years would yield a 95% confidence interval of 4.1 to 11.5% for an annual event rate of 7%.

An additional analysis will be done to compare the incidence of vascular events between the two treatment groups. This analysis will be based on the intention-to-treat principle and reported in terms of the hazard ratio with corresponding 95% confidence intervals calculated with the Cox proportional hazard model.

We consider the precision of the above estimates sufficient for reliable calculation of the sample size of a conclusive randomised clinical trial.

### Safety and indemnity

The major risk of endovascular treatment is dislodgement and distal embolisation of plaque and thrombotic debris, which may lead to stroke. In a recent review of reports on 331 endovascular treatments of VB artery stenosis, the 30-day risk of TIA, stroke, or death was 6.4% [6]. In another review of stenting procedures for vertebral or basilar artery stenosis reported up to 2005, the periprocedural risk of stroke or death was 1.6% for proximal vertebral artery stenosis and 13.8% for distal artery stenosis [7]. Other neurological complications, such as vertebral artery dissection, occurred in 2.6% and 6.0% of the cases, respectively, and non-neurological complications, such as inguinal haematoma, in 1.3% and 2.8%. As mentioned above, the complication rates for distal VB stenting do probably not apply to stenting of the distal vertebral artery outside the acute phase of posterior circulation ischaemia. After a mean follow-up of one year, restenosis has been reported to occur in one quarter of the cases of proximal vertebral artery stenting. However, case series are prone to publication bias and the percentages reported may be different in daily clinical practice. In all randomised trials on carotid stenting versus CEA, the 30-day incidence of any stroke or death was 8.2% (126/1492) after stenting [8]. Moreover, a limitation in interpreting data on vertebral artery stenting is the absence of data describing the risk of recurrent stroke in patients with vertebral stenosis on best medical therapy alone.

### Ethical considerations

The study will be conducted according to the principles of the Declaration of Helsinki, as amended in 2000 and clarified in 2004, and in accordance with the Dutch Medical Research Involving Human Subjects Act (WMO). Approval by the local medical ethical review board is required for each participating centre before start of patient inclusion. The patients will be informed about the trial by their treating physician but will whenever possible be asked for consent by a physician not involved in their treatment. Patients should reach a decision about participation within 180 days after the last vertebrobasilar symptom.

The trial compares two existing forms of treatment currently used in many hospitals worldwide. The investigators anticipate that some patients may be harmed inadvertently as a result of either the stenting procedure or the decision to refrain from invasive treatment. The determination of the rate of these adverse outcome events is in fact the major aim of the trial. In the majority of patients with a recently symptomatic carotid stenosis of 50% or greater, CEA reduces the long-term risk of stroke despite the risks of the intervention [5,14] but at present it is unknown whether this also applies to stenting of vertebral artery stenosis. The trial protocol does not subject patients to hazards that would not have been encountered if they had received the trial treatments outside the context of the trial in routine clinical practice, except for the very small chance of a complication of CTA performed one year after start of treatment, such as an allergic reaction or contrast nephropathy. Individual investigators and hospitals are required to take responsibility for the occurrence of any adverse events in the same way as they would do if the treatments were performed outside the trial.

The sponsor/investigator has a liability insurance which is in accordance with article 7, subsection 6 of the WMO.

The sponsor (also) has an insurance which is in accordance with the legal requirements in the Netherlands (Article 7 WMO and the Measure regarding Compulsory Insurance for Clinical Research in Humans of 23 June 2003). This insurance provides coverage for damage to research subjects through injury or death caused by the study.

### Adverse event reporting

An *adverse event *is any unfavourable and unintended sign, symptom, or disease occurring during the follow-up period of the study. Adverse events occurring after randomisation will be recorded on the adverse event page of the CRF.

A *serious adverse event *is defined as any adverse event that results in:

1. death;

2. a life-threatening condition;

3. inpatient hospitalisation or prolongation of existing hospitalisation;

4. persistent or significant disability/incapacity.

An *important medical event *that may not result in one of the above conditions may be considered a serious adverse event when, based upon medical judgement, it may jeopardise the patient and may require medical or surgical intervention to prevent one of the outcomes above. A *reasonably related adverse event *is defined as one that is possibly, probably, or definitely related to the trial treatment.

All serious adverse events occurring within 30 days after stenting (or within 30 days after randomisation in the conservative treatment group) and adverse events that are serious and reasonably related to the trial treatment but occur after the first 30 days require completion of the safety report, which should be sent to the trial co-ordination centre within 5 working days of observation or notification of the event. All serious adverse events not related to trial treatment during follow-up require completion of the safety report at the planned follow-up visits.

Each outcome event will be reported to the Data Monitoring and Safety Committee (DMSC). We propose the DMSC to use the lower limit of the 95% confidence interval of the complication rate as a criterion to advice the Steering Committee to stop the trial. This lower limit should not be higher than 8.2%, the rate reported for carotid artery stenting. Moreover, we will ask the DMSC to perform one interim analysis halfway during the trial, i.e. after one half of the planned patient-years have been accrued. For this interim analysis we propose the simple 3 standard deviation stopping rule proposed by Peto, that essentially advises to stop at p < 0.001 [16]. We consider it highly unlikely, however, that this threshold will be reached in our relatively small feasibility study.

### Publication of the trial results

The trial results will be published by the members of the Steering Committee, on behalf of the investigators.

## Discussion

We present the protocol of a randomised clinical trial designed to test the safety and feasibility of vertebral artery stenting in patients with a symptomatic stenosis ≥ 50% of a vertebral artery. Case reports suggest that vertebral artery stenting is relatively safe with a periprocedural risk of stroke or death ranging from 1.6 to 13.8%.

In a recent review, stenting of the extracranial vertebral artery in 313 patients resulted in technical success in 98 to 100% of the cases, with a 0.3% risk of death and 5.5% risk of neurological complications [7]. Stenting of the distal (intracranial) vertebral artery in 283 patients was associated with technical success in 97 to 98%, but with higher complication rates: a case fatality rate of 3.2% and a 17.3% risk of neurologic complications. The differences in complication rates of stenting between the proximal and distal vertebral artery may be explained by the fact that stenting of the distal vertebral artery was more frequently performed in the acute phase of vertebral or basilar occlusion and was considered technically more difficult.

The interpretation of outcome after vertebral stenting is complicated by the lack of data on the risk of recurrent stroke in patients with vertebral stenosis on best medical therapy alone. To date no reliable data are available on the prognosis of VB stroke. In a recent review, the risk on recurrent stroke in the acute phase (up to seven days) is considered probably higher in patients presenting with a VB event compared with patients presenting with carotid events [1].

Stenting of the proximal vertebral artery might be associated with a relatively high rate of restenosis at follow-up. In the above-mentioned review, restenosis of the proximal vertebral artery has been reported in a quarter of the cases after a mean follow-up of 11.8 months [7]. The definition of restenosis varied for the different studies. Detection criteria included maximal flow velocity in the stenosis in relation to prestenotic or poststenotic segments, pulsatility, anterograde or retrograde direction of flow, and the presence of end-diastolic flow signal proximal to the lesion on duplex ultrasound. Restenosis in the distal vertebral artery was detected in about one fifth of the cases after a mean follow-up of 7.5 months. Most of the patients with restenosis remained asymptomatic. It has been suggested that neointimal proliferation following stent placement has a lower risk on thromboembolic events than atherosclerosis [17]. Possible predictors for restenosis are high age, low pre-treatment vessel diameter and post-treatment stenosis [7,18,19].

Since the natural course of a symptomatic vertebral artery stenosis in patients on best medical treatment alone is unknown and the exact indications for vertebral stenting are unclear a randomised clinical trial is needed. By comparing outcome in patients on best medical treatment alone and patients with vertebral artery stenting, a conclusion can be drawn on the safety and feasibility of vertebral artery stenting in symptomatic vertebral artery stenosis.

Conventional angiography is the gold standard for the diagnosis of vertebral artery stenosis. Randomisation to either stenting or best medical treatment in this study will take place before angiography is performed. It is not considered ethical to perform conventional angiography in each patient included in the study because this procedure is associated with a small but inevitable risk of stroke. This might lead to randomisation of patients to stenting without a significant stenosis on conventional angiography. In approximately half of the patients (patients randomised to best medical treatment alone), grading of the stenosis will be done on the basis of CTA or MRA and duplex ultrasound alone. Studies of sufficient quality validating the accuracy of diagnosing and grading vertebral artery stenosis with non-invasive imaging techniques against the gold standard of intra-arterial angiography are scarce. No studies have compared the different imaging modalities against intra-arterial angiography in the same cohort of patients. Contrast-enhanced MRA and possibly CTA may be more sensitive in diagnosing vertebral artery stenosis than duplex ultrasound [20].

The trial has started 1 June 2008 in two centres in the Netherlands. Four other Dutch centres are expected to join the trial shortly. Other centres, also from other countries than the Netherlands, that have adequate experience with the management of vertebral artery stenting are welcome to participate.

## Trial organisation

### Steering committee

The Steering Committee carries the ultimate responsibility for the trial. Specific tasks of the steering Committee are:

1. approval of the study protocol;

2. approval of amendments to the study protocol;

3. deciding whether or not to continue the trial based on the recommendations of the DMSC;

4. reviewing protocols for satellite studies;

5. approval of reports and publications of the trial.

The Steering Committee is constituted of the principal investigators of each actively randomising centre, and of the members of the executive committee.

As of 20 July 2008, members of the Steering Committee are (in alphabetical order): A. Algra,* clinical epidemiologist, University Medical Centre, Utrecht – A. Compter,* research physician, University Medical Centre, Utrecht – L.J. Kappelle,* neurologist, University Medical Centre, Utrecht, co-principal investigator – T.H. Lo, interventional radiologist, University Medical Centre, Utrecht – W.P.Th.M. Mali, radiologist, University Medical Centre, Utrecht – F.L. Moll, vascular surgeon, University Medical Centre, Utrecht – W.J. Schonewille,* neurologist, St. Antonius Hospital, Nieuwegein – J.A. Vos,* radiologist, St. Antonius Hospital, Nieuwegein – H.B. van der Worp,* neurologist, University Medical Centre, Utrecht, co-principal investigator. The members of the Executive Committee, who are responsible for the trial on a day-to-day basis, are marked with an asterisk.

### Trial Coordination Centre

The Trial Co-ordination Centre is located at the Trial Office Neurology of the Department of Neurology of the University Medical Centre, Utrecht, The Netherlands. The centre will be staffed by the trial co-ordinator and a data manager.

### Data Monitoring and Safety Committee

The DMSC performs analyses of the unblinded data on a permanent basis and formulates recommendations for the Steering Committee on the continuation of the trial. The Data Monitoring Committee may also offer unsolicited recommendations.

Members of the Data Monitoring Committee are being sought.

## Competing interests

The authors declare that they have no competing interests.

## Authors' contributions

AC participated in writing the protocol and is concerned with patient recruitment and data management. HBW wrote the protocol, applied for financial support, and is concerned with patient recruitment. WJS participated in writing the protocol and is concerned with patient recruitment. AA participated in writing the protocol and is responsible for data management. THL participated in writing the protocol and performs stenting. WPThMM participated in writing the protocol. FLM participated in writing the protocol. JAV participated in writing the protocol and performs stenting. LJK participated in writing the protocol, applied for financial support and is concerned with patient recruitment. All authors have read and approved the manuscript.

## Appendix. definitions of vascular complications

1. Death from vascular causes:

a. Fatal cerebral infarction: cerebral infarction causing an increase of handicap to Rankin 4 or 5, followed by death. Death should have been an unlikely event without the preceding infarction.

b. Fatal cerebral hemorrhage: cerebral haemorrhage causing an increase of handicap to Rankin 4 or 5, followed by death. Death should have been an unlikely event without the preceding bleeding.

c. Fatal myocardial infarction: Documented myocardial infarction (see 6) followed by death, at least one hour after onset of symptoms.

d. Definite sudden death: witnessed sudden death with reliable observation of timing; i.e. patient died within one hour after onset of symptoms.

e. Probable sudden death: witnessed death, but unreliable data on timing of events, or found dead and previously "healthy."

f. Terminal heart failure.

g. Fatal arterial bleeding.

h. Other fatal vascular complication, e.g. gastric bleeding, pulmonary embolism.

2. Cerebral infarction.

a. Definite new cerebral infarction: clinical evidence of the sudden onset of a new neurological deficit, persisting for more than 24 hours, with a corresponding new infarct on a repeat CT scan.

b. Probable new cerebral infarction: clinical evidence of the sudden onset of a new neurological deficit, or an increase in an existing deficit, persisting for more than 24 hours, without a new infarct on repeat CT scan.

3. Intracerebral haemorrhage

a. Clinical evidence of a sudden new neurological deficit, or an increase in an existing deficit, persisting for more than 24 hours, with a corresponding cerebral haemorrhage on a CT or MRI scan.

4. Subarachnoid haemorrhage

a. A sudden unusual headache and/or a reduction in consciousness with a corresponding subarachnoid haemorrhage on CT or MRI, or signs of subarachnoid haemorrhage in the cerebrospinal fluid.

5. Unspecified stroke

a. Clinical evidence of a sudden new neurological deficit, or an increase in an existing deficit, persisting for more than 24 hours, with no imaging performed.

6. Myocardial infarction

a. Myocardial infarction: At least two of the following characteristics have to be documented: a history of chest discomfort for at least half an hour, specific cardiac enzymes more than twice the upper limit of normal, or the development of specific abnormalities (e.g. Q waves) on the standard 12-lead electrocardiogram.
